# Supplementary material for: iProtDNA-SMOTE: Enhancing protein-DNA binding sites prediction through imbalanced graph neural networks
Source: PLoS One. 2025 May 13;20(5):e0320817. doi: 10.1371/journal.pone.0320817 (PMC12074593; doi:10.1371/journal.pone.0320817)
Supplement: S1 Tables — (DOCX) [file pone.0320817.s001.docx]

**Supplementary Tables**

**Table 1. Performance Comparison of Various Methods Across Different Datasets.**

| Method | Testing Dataset | Spe | Rec | Pre | F1 | MCC | AUC |
| --- | --- | --- | --- | --- | --- | --- | --- |
| DRNAPred | 46 | 0.692 | 0.677 | 0.185 | 0.291 | 0.226 | 0.755 |
| DRNAPred | 129 | 0.937 | 0.233 | 0.190 | 0.210 | 0.155 | 0.693 |
| DNAPred | 46 | 0.655 | 0.671 | 0.157 | 0.254 | 0.194 | 0.730 |
| DNAPred | 129 | 0.954 | 0.396 | 0.353 | 0.373 | 0.332 | 0.845 |
| DNAPred | 181 | 0.948 | 0.334 | 0.223 | 0.267 | 0.233 | 0.802 |
| SVMnuc | 46 | 0.666 | 0.668 | 0.154 | 0.250 | 0.192 | 0.715 |
| SVMnuc | 129 | 0.966 | 0.316 | 0.371 | 0.341 | 0.304 | 0.812 |
| SVMnuc | 181 | 0.960 | 0.289 | 0.242 | 0.263 | 0.229 | 0.803 |
| NCBRPred | 46 | 0.674 | 0.677 | 0.165 | 0.265 | 0.207 | 0.713 |
| NCBRPred | 129 | 0.969 | 0.312 | 0.392 | 0.347 | 0.313 | 0.823 |
| NCBRPred | 181 | 0.964 | 0.259 | 0.241 | 0.250 | 0.215 | 0.771 |
| DBPred | 46 | 0.784 | 0.708 | 0.243 | 0.362 | 0.320 | 0.794 |
| GraphBind | 129 | - | 0.676 | 0.425 | 0.522 | 0.499 | 0.927 |
| GraphBind | 181 | 0.933 | 0.624 | 0.293 | 0.399 | 0.392 | 0.904 |
| CLAPE-DB | 46 | 0.835 | 0.747 | 0.306 | 0.434 | 0.401 | 0.871 |
| CLAPE-DB | 129 | 0.955 | 0.464 | 0.396 | 0.427 | 0.389 | 0.881 |
| CLAPE-DB | 181 | 0.931 | 0.413 | 0.212 | 0.280 | 0.252 | 0.824 |
| iProtDNA-SMOTE | 46 | 0.963 | 0.438 | 0.532 | 0.481 | 0.438 | 0.875 |
| iProtDNA-SMOTE | 129 | 0.972 | 0.442 | 0.497 | 0.468 | 0.437 | 0.896 |
| iProtDNA-SMOTE | 181 | 0.982 | 0.244 | 0.372 | 0.294 | 0.276 | 0.874 |
