# Supplementary material for: iProtDNA-SMOTE: Enhancing protein-DNA binding sites prediction through imbalanced graph neural networks
Source: PLoS One. 2025 May 13;20(5):e0320817. doi: 10.1371/journal.pone.0320817 (PMC12074593; doi:10.1371/journal.pone.0320817)
Supplement: Attachment — Submitted filename: Review Comments iPROTDNA-smote.docx [file pone.0320817.s006.docx]

Review Comments iPROTDNA-SMOTE

1. The manuscript studies an important area in understanding the biological process and the cellular functions arising out of these interactions.
2. The manuscript is well written, the literature is thoroughly reviewed and the study has been organized in a systematic fashion.
3. The language of the manuscript is good, but needs a little proof reading to fix some language and grammatical errors.
4. The	working of ESM2 and Graph SMOTE should have been elaborated within the manuscript, so that it becomes easy for the reader to understand the class balancing and the embeddings generated by ESM2. Although the raw data files on GitHub contain sequence and encoding, but the graph data and ESM2 embedding are in binary format which is beyond comprehension. It would be beneficial for this study to explain the output of ESM2 and the graph structure derived from such embeddings.
5. The authors are also advised to perform some downstream analysis for the novel predictions generated by their model if any to show its relevance in predicting biological functions associated with this DNA binding protein.
