# Supplementary material for: iProtDNA-SMOTE: Enhancing protein-DNA binding sites prediction through imbalanced graph neural networks
Source: PLoS One. 2025 May 13;20(5):e0320817. doi: 10.1371/journal.pone.0320817 (PMC12074593; doi:10.1371/journal.pone.0320817)
Supplement: Attachment — Submitted filename: comments.docx [file pone.0320817.s007.docx]

The article was reviewed under the title " iProtDNA-SMOTE: Enhancing Protein-DNA Binding Sites Prediction through Imbalanced Graph Neural Networks".

Overall, the focus of the study is good. However, I would like to offer several recommendations that authors may find useful in the process of revising their manuscript:

1- Given the main structure of the paper, which is based on graph-based neural networks, more previous research needs to be studied.
2- In the classification of Graph structured data section, graph convolution operations need to be discussed further.
3- Also in the classification of Graph structured data section, more explanation should be provided about collecting neighbor features.
4- In Table 1, the training and test datasets are different. Please explain why this is done.
