# Supplementary material for: iProtDNA-SMOTE: Enhancing protein-DNA binding sites prediction through imbalanced graph neural networks
Source: PLoS One. 2025 May 13;20(5):e0320817. doi: 10.1371/journal.pone.0320817 (PMC12074593; doi:10.1371/journal.pone.0320817)
Supplement: Attachment — Submitted filename: Suggestions for Improvement.docx [file pone.0320817.s008.docx]

**Suggestions for Improvement**

1. **Sensitivity Analysis**:

Include experiments to analyze the trade-offs between precision and recall for various datasets, particularly focusing on the biological implications of missing DNA-binding residues.

2. **Efficiency Metrics**:

Provide a comparison of computational time and resource utilization against competing methods to offer a holistic evaluation of the model’s practicality.

3. **Future Directions**:

Discuss potential integrations with advanced graph attention mechanisms or hybrid models to address current limitations in handling long protein sequences.

Consider incorporating more diverse datasets or synthetic benchmarks to evaluate robustness further.

4. **Error Analysis**:

A deeper error analysis to identify specific cases where the model underperforms (e.g., specific protein classes or sequence patterns) would provide actionable insights for further refinement.
