# Supplementary material for: iProtDNA-SMOTE: Enhancing protein-DNA binding sites prediction through imbalanced graph neural networks
Source: PLoS One. 2025 May 13;20(5):e0320817. doi: 10.1371/journal.pone.0320817 (PMC12074593; doi:10.1371/journal.pone.0320817)
Supplement: Attachment — Submitted filename: comments_declet_1.docx [file pone.0320817.s010.docx]

The article titled " iProtDNA-SMOTE: Enhancing Protein-DNA Binding Sites Prediction through

Imbalanced Graph Neural Networks" was reviewed again.

This revision has significantly improved the manuscript. The authors have satisfactorily addressed my concerns.
